# Behaviour is more important than thermal performance for an Arctic host–parasite system under climate change

**DOI:** 10.1098/rsos.220060

**Published:** 2022-08-24

**Authors:** Stephanie J. Peacock, Susan J. Kutz, Bryanne M. Hoar, Péter K. Molnár

**Affiliations:** ^1^ Department of Ecosystem and Public Health, University of Calgary, 3280 Hospital Drive NW, Calgary, AB Canada, T2N 4Z6; ^2^ Department of Biological Sciences, University of Toronto Scarborough, 1265 Military Trail, Toronto, ON Canada, M1C 1A4; ^3^ Department of Ecology and Evolutionary Biology, University of Toronto, 25 Willcocks Street, Toronto, ON Canada, M5S 3B2

**Keywords:** parasite, migratory escape, population dynamics, arrested development, metabolic theory, partial-differential equation

## Abstract

Climate change is affecting Arctic ecosystems, including parasites. Predicting outcomes for host–parasite systems is challenging due to the complexity of multi-species interactions and the numerous, interacting pathways by which climate change can alter dynamics. Increasing temperatures may lead to faster development of free-living parasite stages but also higher mortality. Interactions between behavioural plasticity of hosts and parasites will also influence transmission processes. We combined laboratory experiments and population modelling to understand the impacts of changing temperatures on barren-ground caribou (*Rangifer tarandus*) and their common helminth (*Ostertagia gruehneri*). We experimentally determined the thermal performance curves for mortality and development of free-living parasite stages and applied them in a spatial host–parasite model that also included behaviour of the parasite (propensity for arrested development in the host) and host (long-distance migration). Sensitivity analyses showed that thermal responses had less of an impact on simulated parasite burdens than expected, and the effect differed depending on parasite behaviour. The propensity for arrested development and host migration led to distinct spatio-temporal patterns in infection. These results emphasize the importance of considering behaviour—and behavioural plasticity—when projecting climate-change impacts on host–parasite systems.

## 1. Introduction

Annual mean temperature over northern Canada increased by 2.3°C from 1948 to 2016, roughly three times the mean global warming rate [1]. Over the next century, this warming trend is expected to continue, with Canada's annual mean temperature projected to increase as much as 6°C and more extreme changes in the winter and in the Arctic [2]. Rising temperatures have dramatic effects in the Arctic, where thawing of the cryosphere can lead to positive feedbacks, accelerated warming and the transformation of entire ecosystems [3,4]. The direct impact of these changes on high-profile Arctic wildlife such as polar bears has received considerable attention from both scientific and public spheres (e.g. [5,6]), but the implications for microfauna, including parasites, are less well understood (but see [7,8]).

Host–parasite dynamics are complex multi-species interactions that often involve environmentally sensitive components (e.g. vectors, free-living stages and ectoparasites). Initial predictions for how climate change would affect disease risk assumed that parasite development rates would increase with temperature, consistent with the metabolic theory of ecology (MTE) [9,10], and thus, accelerate parasite transmission and increase disease risk [11,12]. These predictions have, in some cases, been borne out. For example, climate change has shortened the generation time of the arctic protostrongylid *Umingmakstrongylus pallikuukensis* (a lung nematode of muskoxen) from 2 years to 1 year [12], and these changes have played a key facilitative role in the recently observed range expansion of this parasite [13,14]. However, mortality of free-living parasite stages is also predicted to increase with warming temperatures ([15], e.g. [16]), requiring approaches that allow considering how multiple, interacting traits, each with their own thermal sensitivity, shape resulting host–parasite dynamics [10,17]. The net effect on *R*_0_, the basic reproduction number of a pathogen or parasite population, also depends on where the current and projected temperatures lie within the parasite's locally adapted thermal niche (e.g. [18]). Any realized benefit (or cost) to the parasite population from climate change will also depend on the distribution and abundance of hosts, vectors and/or intermediate hosts, which are themselves shifting with climate change [19].

Parasites may also adapt to a changing climate by changing their behaviour or life-history strategy (e.g. [20,21]). Parasites have evolved unique traits to increase survival and transmission within highly variable environments. For example, dormancy can allow hardy parasite stages to persist inside or outside the host with relatively low metabolic costs when environmental conditions are unfavourable for development. Dormancy can occur for a set period of time (diapause) or until the environment is suitable for development (quiescence; [22,23]). Arrested development of within-host parasite stages can avoid wasting energy on reproduction when eggs would not survive in the environment and can synchronize egg shedding during periods favourable for transmission [24]. The proportion of parasites entering dormancy and the time of year this happens may differ between temperate and tropical environments. For example, in northern latitudes, parasites may be dormant overwinter to avoid shedding eggs during extreme cold, whereas parasites in southern latitudes may be dormant in the heat of the summer to avoid desiccation and heat-induced mortality of free-living stages. For some parasites, the propensity for dormancy is an inherited trait with the potential for adaptive evolution [25]. Latitudinal variation in life-history strategies may also be the result of phenotypic plasticity. Either way, we might expect that the proportion of parasites undergoing arrested development may change in response to a warming climate [26].

Climate change is having cascading effects in Arctic ecosystems [4] with the potential to affect wildlife health [26]. Understanding and preparing for these changes requires consideration of how the above physiological and behavioural responses of hosts and their parasites will interact to affect host–parasite dynamics [10]. Arctic species are ideal model systems to study these effects because they have evolved under severe climatic constraints and are thus sensitive to shifts in the timing and magnitude of seasonal temperature changes [12]. Here, we present a case study of how climate change interacts with host migration, parasite dormancy and parasite thermal responses, to influence the population dynamics of barren-ground caribou (*Rangifer tarandus* ssp.) and their common nematode parasite *Ostertagia gruehneri*.

## 2. Study system

Barren-ground caribou are a sub-species of caribou. They are known to undertake the longest migrations of any land mammal [27], from overwintering ranges in the taiga forests to calving grounds on the tundra. Barren-ground caribou herds undergo long-term cycles in abundance [28,29], but most populations have been declining in recent decades [30,31]. These declines have prompted concerns over the current and future impacts of climate changes on caribou [32].

*Ostertagia gruehneri* is a common gastrointestinal nematode parasite of caribou and reindeer. It can cause decreased food intake, weight loss and reduced pregnancy rates in heavily infected hosts [33–35]. Through its effects on fecundity, it has been shown to regulate populations of Svalbard reindeer [36]—one of the few examples of parasite regulation of a wildlife host population.

**Figure 1.**
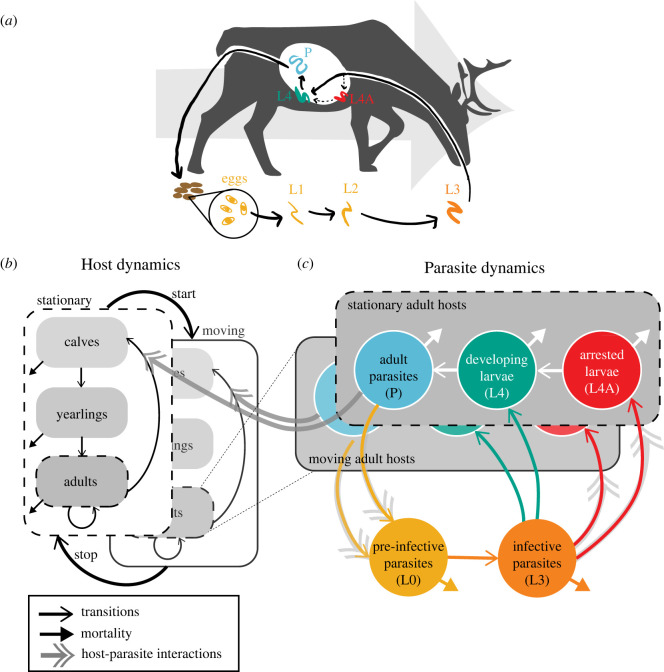
Illustration of (*a*) the caribou host–*O. gruehneri* parasite system and (*b,c*) the mathematical model that we developed to represent the population dynamics. The model includes three host stages (calves, yearlings and adults; (*b*)), but models parasite dynamics in adult hosts only (dotted lines from (*b*) to (*c*)). The parasite populations (*c*) include three within-host stages (arrested larvae, developing larvae and adult parasites) and two free-living stages in the environment (pre-infective and infective). Host and parasite populations interact via density-dependent environmental transmission (double-headed arrows) and parasites affect host population dynamics through negative impacts on host reproduction (grey double-headed arrows). The pre-infective parasite stage (L0 in (*b*)) combines eggs, L1, and L2 larvae (*c*).

*Ostertagia gruehneri* has a direct life cycle: adult parasites reproduce sexually within the abomasum (fourth stomach) of their ruminant hosts; eggs are shed into the environment in hosts' faeces and develop through pre-infective L1 and L2 stages to infective L3, which are then inadvertently ingested by hosts while grazing and develop through L4 and adult stages (figure 1*a*). Ingested larvae can arrest their development at the L4 stage within the mucosa of the abomasum, with emergence from the mucosa and development to adult parasites resuming synchronously in the spring [24].

In the central Canadian Arctic, the proportion of larvae that arrest approaches 100%, which is thought to improve transmission success in a highly seasonal environment with a migratory host population [24,37]. In this region, the life cycle of *O. gruehneri* may take 2 years because (i) larvae ingested Year 1 arrest their development and do not mature to adults until the following spring (Year 2) and (ii) eggs shed by these adults must undergo a temperature-dependent development in the environment to the infective L3 stage, by which time, hosts have migrated away from the contaminated environment (table 1). Free-living larval stages of *O. gruehneri* have evolved to tolerate freezing in order to overwinter and infect hosts when they return to calving and summer ranges in Year 2 [38]. There is anecdotal evidence that a shift to warmer climes reduces the propensity for larvae to arrest in their hosts (i.e. they instead develop immediately to reproductive adults), shortening the life cycle by allowing ingested larvae to develop and reproduce within a single summer [24]. In addition to potential impacts of climate on life-history strategy, the development and mortality rates of free-living parasite stages are highly dependent on temperature [10,39,40], with development and survival rates peaking at different thermal optima [16].

**Table 1.** Summary of the phenology of *O. gruehneri* life cycles with arrested and direct development. Refer to figure 1*a* for illustration of the parasite life cycle.

year	season	arrested development	direct development (cold)	direct development (warm)
Year 1	spring	*L3 ingested*	*L3 ingested*	*L3 ingested*
	summer	*L3 ingested,* L4 arrest in hosts	*L3 ingested,* develop to adults, shed eggs	*L3 ingested,* develop to adults, shed eggs
	autumn/winter	L4 arrest in hosts, hosts migrate away	eggs don't develop to L3 before *hosts migrate away*	eggs develop to L3 (could also happen in summer)^a^, *next generation L3 ingested* before *hosts migrate away*
Year 2	spring	hosts return on spring migration, L4 resume development, develop to adults, lay eggs	hosts return on spring migration, *next generation L3 ingested*	
	summer	develop to adults, lay eggs		
	autumn/winter	*hosts migrate away,* larvae develop^a^		
Year 3	spring	*next generation L3 ingested*		

^a^Temperature dependent.

To understand the net outcome of climate change on this system, we must consider the effects of temperature on the performance of parasite larvae in light of the temporal and spatial patterns of host availability and the propensity of larvae to arrest. To simulate the spatio-temporal transience of host–parasite overlap, we adapted and applied a host–parasite population model that included the spatial movement of hosts along a migration corridor [41,42]. In model simulations, we considered different scenarios for behavioural strategies of the parasite and the host: the proportion of ingested larvae that underwent arrested development (as opposed to direct development) and the movement (or not) of host populations along a circular migration corridor, respectively.

## 3. Methods

We combined laboratory experiments and simulations of a host–parasite population model to estimate how the caribou–*O. gruehneri* host–parasite system may be affected by climate change. First, we implemented several temperature-controlled experiments to test the thermal tolerance of free-living *O. gruehneri* stages. To understand how these thermal responses affect host–parasite population dynamics, we then included the estimated temperature dependencies of development and mortality in a mechanistic host–parasite population model. Data and relevant code for this research work are stored in GitHub: https://github.com/sjpeacock/OsterBou-pop and have been archived within the Zenodo repository: https://doi.org/10.5281/zenodo.6637012.

### 3.1. Laboratory experiments

We infected captive reindeer (*Rangifer tarandus tarandus)* with *O. gruehneri* third-stage larvae (L3) sourced from the Bathurst caribou herd, Northwest Territories (NT) and Nunavut (NU), Canada [38]. *Ostertagia gruehneri* eggs were isolated from reindeer faeces [24,43] and randomly assigned to one of 40 Petri dishes (3 cm diameter) at a density of approximately 50 eggs per dish. Petri dishes were then distributed into incubators with 100% relative humidity and set at 5, 10, 15, 20, 25, 30, 35 and 40°C, respectively, yielding five replicate cohorts for each temperature trial. We examined Petri dishes daily and recorded the number and development stage of live individuals (eggs, L1, L2 and L3; Hoar *et al*. [38]). Examination of all replicates within a given temperature was reduced to once every 3 days after one L3 was observed in each of the five replicates, with the exception of 5°C when a frequency of 3 days was maintained from the start of the experiment due to slower development times at that temperature. At each sampling occasion, we performed egg and larval counts three times for every replicate dish to be able to quantify observation error in detecting eggs and larvae, in correctly identifying the developmental stages of larvae and in correctly assessing larval survival. Replicates continued to be examined until no live eggs or larvae were observed, or for a maximum of 120 days (a conservative estimate of the number of days with mean temperatures greater than 0°C in the Canadian low Arctic; Russell *et al*. [44]).

To estimate development and mortality rates of free-living stages, we developed a cohort model that tracks pre-infective (egg, L1 and L2) and infective (L3) stages through time. As we are primarily interested in the appearance of the infective L3, the model considers a simplified life cycle where eggs, L1 and L2 are pooled into a pre-infective class (subscript 0) and larvae in the L3 stage constitute the infective class (subscript 3) [16,45]. At temperature *T*, the number of pre-infectives that are alive but not yet developed to the infective stage on day *t* depends on the pre-infective mortality rate, *μ*_0_(*T*) (d^−1^), and the expected development time, *ρ*_0_(*T*)^−1^, where *ρ*_0_(*T*) (d^−1^) is the expected development rate from egg to L3 at temperature *T*. We assumed a lognormal distribution of development times among individuals, with an s.d. *σ* [46]. We assumed that *σ* was constant across temperatures because there is no theoretical basis for temperature dependence of this parameter [47].

The expected number of individuals that start the L3 stage exactly at time *t* is obtained from the cumulative probability density function of the lognormal development distribution, discounted for any mortality occurring in the pre-infective stage. The number of infective larvae alive at time *t* is then calculated by integrating over all larvae that have reached the L3 stage before time *t*, discounting for mortality that has occurred since these larvae started the L3 stage at rate *μ*_3_(*T*) (d^−1^). Model equations for the expected number of pre-infective and infective parasites are provided in the electronic supplementary material, appendix A.

We assessed up to four different sub-models for the temperature dependencies of *μ*_0_(*T*), *ρ*_0_(*T*) and *μ*_3_(*T*): (i) no parametric relationship among rates at different temperatures, (ii) constant rates across temperatures, (iii) an Arrhenius relationship that describes increasing rates with temperature [48], or (iv) a Sharpe–Schoolfield relationship that builds on the Arrhenius relationship to include the possibility of upper and/or lower temperature thresholds [16,49].

We fit the cohort model, incorporating temperature-dependent parameters *μ*_0_(*T*), *ρ*_0_(*T*) and *μ*_3_(*T*) in a Bayesian framework that could accommodate a hierarchical structure accounting for potential variability in parameters among replicates, latent variables, as well as the triplicate counts at each sampling occasion. We included a random effect for replicate (i.e. Petri dish) on the parameters *μ*_0_(*T_i_*), *ρ*_0_(*T_i_*), *μ*_3_(*T_i_*) and *σ* within each temperature trial *i*. Due to counting error, the true initial number of eggs in each Petri dish was not known and so it was treated as a latent variable to be estimated. The likelihood of the pre-infective and infective counts from day 1 to day 120 was calculated assuming Poisson count error for the triplicate observations, with an expected count equal to the prediction from the cohort model for the given day, temperature and replicate. Details of the model fitting process are provided in the electronic supplementary material, appendix A.

Testing all possible combinations of five MTE relationships (constant, Arrhenius and Sharpe–Schoolfield with lower, upper or both thresholds) for the three parameters (*n* = 3^5^ = 243 models) was not practically feasible due to the computation time involved and also would have increased the chance of spurious significant results. We chose nine models to test based on initial parameter estimates and limitations of the data (electronic supplementary material, table A2). For example, there was little to no development of larvae to the infective stage at high temperature treatments, and so estimating upper temperature thresholds for a Sharpe–Schoolfield relationship for the mortality rate of infective larvae was difficult. The fits with independent parameters at each temperature (no temperature relationship) showed no increase in mortality or decrease in development at low temperatures, so we did not include a lower temperature bound when fitting the Sharpe–Schoolfield relationship.

In total, we had 2520 unique sampling instances (i.e. temperature, Petri dish and day combinations) with triplicate counts and two larval stages, yielding 15 120 data points. We evaluated models by holding out one of the five replicates when estimating parameters and using this replicate as a validation dataset. The remaining four replicates were used to estimate model parameters (i.e. the training data). We then calculated the likelihood of the validation data given the model fitted to the training data. This was repeated using each of the five replicates as the validation data and then taking the average likelihood over the five different validation replicates. We took the model with the lowest average negative log-likelihood as the best model and re-fitted that model to the entire dataset to yield the best parameter estimates for inference and prediction.

### 3.2. Host–parasite population modelling

We adapted a migratory host–parasite model developed by Peacock *et al*. [41,42] to the caribou–*O. gruehneri* system. The model includes equations for moving hosts, stationary hosts, the average parasite burden within both moving and stationary adult hosts and stationary free-living parasite larvae in the environment. Moving hosts migrate along a one-dimensional domain that represents a migration corridor; although this ignores dispersal of hosts in two dimensions, it is a reasonable simplification for host species that migrate long distances. Hosts can switch between moving and stationary at rates that depend on space and time, allowing for the local densities of hosts to change throughout the year. From year to year, the same migration corridor is conserved in the model, which is a simplification of reality as migration routes may vary slightly. However, this simplification is unlikely to affect host–parasite simulations because the majority of transmission occurs at stop-over sites (calving and summer ranges), which are generally the same for a given herd from year to year [50].

We expanded the original model to differentiate three host stages (calves, yearlings and adults), three within-host parasite stages (arrested larvae, developing larvae and adult parasites) and two free-living parasite stages (pre-infective and infective). Parasite populations are modelled in adults hosts only, as studies of barren-ground caribou have found low prevalence and abundance of *O. gruehneri* in calves and yearlings (B. Hoar 2007–2009, unpublished data from the Bathurst caribou herd, available on request from the Government of Northwest Territories). The final model consists of 14 partial-differential equations for the change in host and parasite variables over space and time. Biological explanations and parametrization of equations for hosts, within-host parasites and free-living parasite larvae are described below, with equations provided in the electronic supplementary material, appendix B.

#### 3.2.1. Hosts

**Figure 2.**
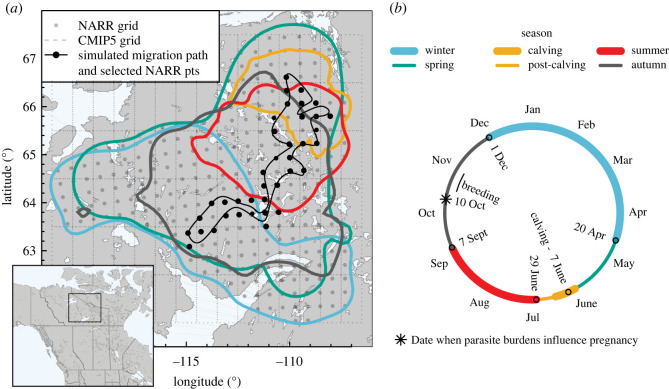
(*a*) Map of the approximate Bathurst caribou ranges (Russell *et al*. [44]; data provided by Don Russell *pers. comm.*) showing a hypothetical one-dimensional migration route through two-dimensional space (thin, black line; see electronic supplementary material, appendix C). The solid dark points are the spatial grid points for ground temperature data used to estimate annual temperature profiles, and the dotted lines are the spatial grid for the CMIP5 projections for temperature change (electronic supplementary material, appendix C). (*b*) Seasonality in the caribou*–O. gruehneri* system. Dates correspond to the average annual cycle for Bathurst caribou [53].

We model the host population dynamics for the Bathurst barren-ground caribou herd, which migrates over a thousand kilometres annually through the NT and NU, Canada (figure 2; [51–53]). Bathurst caribou typically overwinter below the treeline north and east of Great Slave Lake, NT. In late April, they begin their spring migration northward towards calving grounds near Bathurst Inlet, NU. Aggregation of female caribou is high during the calving season, typically lasting two weeks with peak calving around 7 June [51,53]. In the second half of June, the herd undergoes a synchronized, aggregated and directional move away from the calving grounds: the post-calving migration. The summer season (July–August) is characterized by more dispersed grazing over the summer range. In the first week of September, the autumn migration commences with caribou beginning to aggregate for the breeding season in mid to late October [54]. Breeding is followed by the continuation of fall migration south to the winter range.

We captured these general patterns of movement, aggregation and dispersal through three parameters: (i) the migration speed of moving hosts, (ii) the rate at which hosts stop moving, and (iii) the rate at which hosts start moving. For hosts that are moving, we assumed a migration speed of 14 km d^−1^. Although movement speeds can be quite variable among individuals and through time, this is a reasonable average movement speed based on movement rates from GPS-collared female Bathurst caribou [51]. The rates of host stopping and starting were chosen to simulate two periods of host aggregation that may influence parasite transmission: calving to post-calving migration in June, and breeding in October (electronic supplementary material, appendix B). Aggregation was modelled through a spatially and temporally variable stopping rate that forced hosts at the leading edge of the moving population to stop first, allowing the trailing edge of the population to catch up. Conversely, the dispersal of hosts during summer grazing and over winter was modelled by increasing stopping rates first at the trailing edge of the moving population, thereby spreading out host density over the summer or winter range, respectively.

We also considered a ‘non-migratory’ scenario where the population remained on the overwintering grounds by setting the migration speed to 0 km d^−1^. This was to better understand the effects of host migration on parasite dynamics, but also to explore the potential consequences for caribou health if migratory behaviour was lost. The shift to more sedentary behaviour with climate change has occurred in other species [55] and may be driven in caribou by climate-induced changes to their habitats including forage availability and ice conditions [56,57].

Starting host abundance and natural mortality rates of calves, yearlings and adults were informed by demographic modelling of the Bathurst caribou herd [58]. The Bathurst caribou herd has declined since the mid-1980s, with the rate of decline accelerating in recent years. We used parameter estimates for survival of calves (*s_c_* = 0.45 y^−1^), yearlings (*s_Y_* = 0.86 y^−1^) and adults (*s_A_* = 0.86 y^−1^) from 1985, prior to this decline. Daily mortality rates were calculated from these annual survival estimates as *μ_X_* = (1 − *s_X_*)/365 where *X* = *C*, *Y* or *A* for calves, yearlings or adults, respectively.

In the model, host reproduction occurred on 7 June each year (figure 2*b*), when existing caribou moved up one age class, with yearlings joining the adult class and calves becoming yearlings. The number of new calves depended on the number of female caribou arriving at the breeding grounds (prior to the addition of yearlings) multiplied by the proportion of female caribou producing a viable calf. Calving rates are highly dependent on body condition [59,60], which in turn is influenced by parasite burdens [34,35]. Experimental anti-helminthic treatments of Svalbard reindeer have shown a direct link between caribou fecundity and *O. gruehneri* infection the previous October, with a greater positive effect of treatments at higher parasite burdens [36]. We included a decline in the calving rate with increasing October parasite burdens based on the relationship described by Stien *et al*. [33] (electronic supplementary material, appendix B).

We assumed density-dependent transmission, whereby an adult caribou ingests infective parasites at a rate of *β* = 10^−6^ d^−1^ multiplied by the density of infective parasite larvae at the given location and time. The magnitude of the transmission parameter, *β*, is difficult to estimate for wildlife hosts because there are many factors influencing successful transmission. Due to the uncertainty in this parameter, we report the sensitivity of model output to two other levels of transmission in the electronic supplementary material, appendix B: low (*β* = 10^−7^ d^−1^) and high (*β* = 10^−5^ d^−1^).

#### 3.2.2. Within-host parasites

**Figure 3.**
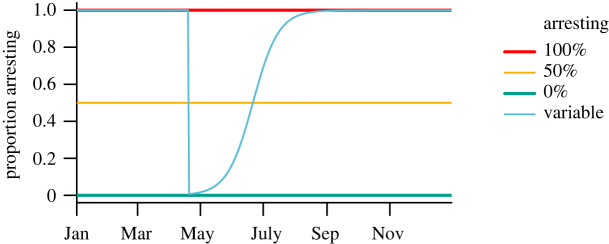
The proportion of ingested larvae that enter arrested development (*y*-axis) as a function of time (*x*-axis) under four scenarios for larval arrested development: (i) 100% arresting (red), (ii) 50% arresting (yellow), (iii) 0% arresting (turquoise), and (iv) variable arresting (blue). The first three scenarios assume the proportion is constant throughout the year, while the fourth scenario models an increase in the proportion from zero at the onset of spring migration to one by the end of the summer, with 50% arresting at the summer solstice (21 June ).

We investigated parasite dynamics under four life-history scenarios, representing different strategies for the arrested development of parasite larvae within hosts (figure 3). The first three scenarios assumed the proportion of larvae that enter an arrested state (*χ*) was fixed throughout the year at 100% arresting (*χ* = 1); a mixed strategy where 50% of larvae arrest (*χ* = 0.5) or 0% arresting (i.e. only direct development; *χ* = 0). The fourth scenario considered an increase in the proportion of larvae that arrest from *χ* = 0 at the onset of the spring migration to *χ* = 1 towards the end of the summer (figure 3). The 100% arresting scenario best represents the current life-history strategy of the parasite [24], while we expect to see a shift towards direct development with climate change based on parasite life-histories in more southern climes [61] and temporal patterns of infection for parasites translocated from the Arctic to southern Alberta [62]. The fourth scenario (variable arresting) may be the most realistic change to occur with a warming climate. In all scenarios, arrested larvae synchronously resumed developing at the onset of spring migration [24].

Mortality and development rates of within-host parasite stages have not been quantified for *O. gruehneri*, so we were forced to look to other systems to inform these parameters. Based on studies of the closely related *O. ostertagi*, a common parasite of domestic cattle, we set the mortality rate of both arrested and developing larvae to *μ*_4_ = 0.002 d^−1^ [63]. The development of ingested larvae to the adult stage can take approximately 17 days [63], so we set the rate at which developing larvae transition to the adult stage to *ρ*_4_ = 0.06 d^−1^. The mortality of adult parasites is density dependent, with increasing mortality at high parasite burdens. We assumed a linear increase in *per capita* adult parasite mortality with parasite burden as parametrized by Grenfell *et al*. [63]: $\mu_{P}=0.1713+0.3082\times 10^{-6}\;\bar{P}$ d^−1^, where $\bar{P}$ is the mean parasite burden per host. In parasite populations that are overdispersed among hosts (as most parasites are [64]), the population-level consequences of density-dependent parasite mortality must consider the distribution of parasites among hosts [65]. We assumed the distribution of parasites among hosts was described by the negative binomial, with fixed overdispersion parameter *q_NB_* = 0.994 that we estimated from parasite surveys of Bathurst caribou [62].

Adult *O. gruehneri* shed eggs at density-dependent rate *λ*(*t*)*P*^1+*γ*^ d^−1^ [66], where *P* is the number of adult parasites per host and *λ*(*t*) is the time-dependent shedding rate that includes annual variability in both parasite egg production and fecal output of hosts (see electronic supplementary material, appendix B), leading to peak egg shedding in the summer months and negligible egg output for November to April [24]. The parameter *γ* < 1 yields lower per-parasite fecundity at high parasite burdens. The parameters that enter into *λ*(*t*) and *γ* were taken from Stien *et al*. [66], who parametrized the fecal egg count model based on observations of *O. gruehneri* infections in Svalbard reindeer.

#### 3.2.3. Free-living parasite larvae

We included temperature-dependent development and mortality of free-living larvae as predicted by the MTE models fit to experimental data, described above. However, because the experiments took place at 5–35°C and did not cover the range of temperatures larvae would be subject to on the tundra (−55–30°C over the Bathurst range [44]), we modified the best-fit relationship in two ways. First, the best-fit MTE model for infective mortality (*μ*_3_) was a constant rate over all temperatures, but we know that mortality must increase at extreme upper temperatures. Therefore, we used parameter estimates for *μ*_3_ from a Sharpe–Schoolfield model with upper bound even though this was not the best-fit model and showed some convergence issues. Second, we included a lower thermal bound on mortality of infective and uninfective larvae. Although there is some evidence that free-living stages can survive over winter [62], we did not have sufficient data on freezing survival of *O. gruehneri* eggs and larvae to estimate lower thresholds for mortality curves. To inform these lower thresholds, we turned to another Arctic-adapted gastrointestinal nematode for guidance: *Marshallagia marshalli*. This parasite has a similar life cycle, developing through L1, L2 and L3 stages in the environment. The lower thermal thresholds for mortality of pre-infective and infective stages of *M. marshalli* were estimated from data at −9, −20 and −35°C in Aleuy *et al*. ([67]; see electronic supplementary material, appendix A for details).

#### 3.2.4. Simulations

We simulated the model over a 200-year period, with starting numbers of calves, yearlings and hosts estimated from the Bathurst herd near its peak in 1985 [58]. We numerically simulated the system of 14 partial-differential equations (electronic supplementary material, appendix B) with a timestep of 1 day on a spatial migration corridor 2270 km long in increments of 2 km. This total migration distance was chosen to correspond to the expected distance moved in a year given the average movement rates during periods of migration [51] and duration of those periods [53]. At each timestep, moving hosts migrated seven grid spaces (14 km d^−1^) while stationary hosts did not change their location. After moving, we applied host and parasite mortality, parasite development, parasite shedding and the switching of hosts between stationary and moving populations. At two points in the year, there were instantaneous changes that took place prior to movement: on 7 June each year, hosts reproduced (see subsection *Hosts*) and on 20 April (coinciding with the onset of spring migration) arrested larvae resumed development.

**Figure 4.**
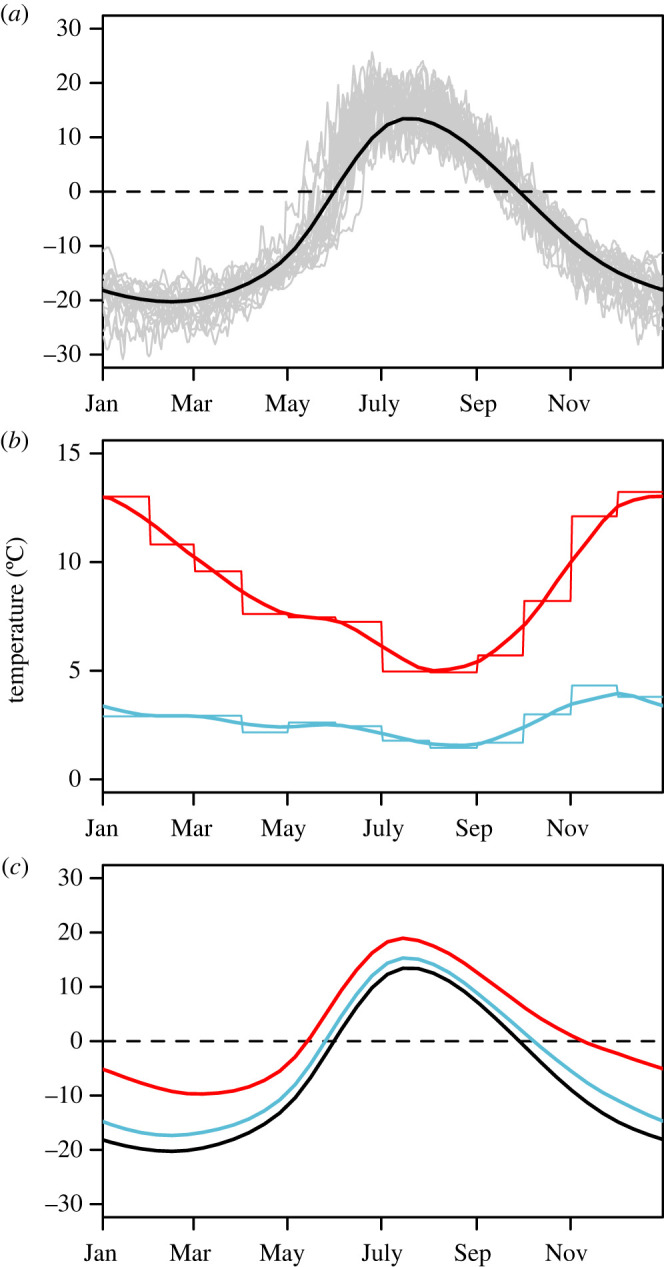
(*a*) Daily mean annual ground temperature for a single location along the migration route (*x* = 660 km on calving grounds), smoothed (black line) over 21 years of data (grey lines). (*b*) The predicted daily increases in air temperature (thick lines) under representative concentration pathway (RCP) 2.6 (blue) and RCP 8.5 (red) emissions scenarios, smoothed from monthly projections (thin line). (*c*) The daily mean temperatures from historical data (black) and under RCP 2.6 (blue) and RCP 8.5 (red) emissions scenarios, calculated as the sum of (*a*) and (*b*).

To understand the potential impact of changing temperatures on parasite dynamics and host health, we considered two different climate-change scenarios superimposed upon the historical annual pattern of ground surface temperatures (figure 4). Ground surface temperatures are more relevant for *O. gruehneri* larvae, which overwinter in the top layers of the soil, than air temperatures. Further, annual patterns in ground temperature may differ substantially from those of air temperatures [68]. Ground temperatures were taken from the North American Regional Reanalysis (NARR) high-resolution spatial projections of soil temperatures at 0 cm depth, provided by the NOAA/OAR/ESRL Physical Sciences Laboratory, Boulder, CO, USA (https://psl.noaa.gov/data/gridded/data.narr.subsurface.html; accessed 16 April 2021). These data are provided in a spatial grid of approximately 32 × 32 km. To summarize the average temperature over the annual migration route of the Bathurst caribou, we simulated a migration path that connected randomly selected points within areas of high historical use for each of the seasonal ranges (winter, calving, summer and autumn; figure 2*a*). We extracted the daily mean ground temperature for grid points along that migration path for the 21-year period from 2000 to 2020 and applied a locally weighted polynomial regression [69,70] to smooth over the annual cycle. The result was a typical annual temperature profile that depended on the location along the migration and the day-of-year (figure 4*a*). We ran simulations over different randomly generated migration paths to ensure our results were not sensitive to the particular spatial points selected (electronic supplementary material, appendix C).

Climate-change scenarios were based on spatially gridded projections of temperature anomalies from the CMIP5 multi-model ensemble [71]. As ground temperature projections were not available, we used the mean monthly air temperature anomalies for the period of 2080–2099 compared with the model's reference period of 1986–2005 for a low emissions scenario (representative concentration pathway (RCP) 2.6) and high emissions scenario (RCP 8.5). Projections were extracted for grid points based on the simulated migration path, as done for the historical ground temperature data. We smoothed the monthly temperature anomalies to yield a smooth curve of projected temperature anomalies for each day of the year and location along the migration route under both emissions scenarios (figure 4*b*). In climate-change simulations, we superimposed this temperature increase onto the annual ground temperature curve (figure 4*c*), abruptly starting in year 101 of the 200-year simulations. This method assumed ground temperature anomalies will mirror air temperature anomalies under climate change (see Discussion).

## 4. Results

### 4.1. Effect of temperature on free-living parasite stages

**Figure 5.**
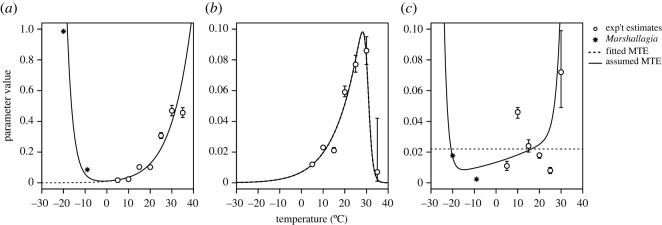
Parameter estimates for (*a*) mortality (d^−1^) of pre-infective parasite stages (eggs, L1 and L2), (*b*) development rate (d^−1^) to infective stage and (*c*) mortality (d^−1^) of infective parasite larvae (L3). Open points are the mean values (±95% credible intervals) estimated independently for each temperature treatment (electronic supplementary material, table A3), while the dotted line is the best-fit MTE relationship fitted to experiments (electronic supplementary material, table A4). For population modelling, we included an upper thermal bound on infective mortality (*c*) even though it was not the best-fitting MTE model and lower thermal bounds on the mortality rates (solid lines; electronic supplementary material, table A5) that could not be estimated from experiments but were informed by studies of freezing survival of *Marshallagia* larvae (asterisks; Aleuy *et al*. [67]).

The mortality rates of both pre-infective (*μ*_0_) and infective (*μ*_3_) parasite stages tended to increase with temperature (figure 5*a,c*). When parameters were estimated independently for each temperature treatment, pre-infective mortality was lowest at 5°C and highest at 30°C (figure 5*a*; see electronic supplementary material, table A3 for specific parameter estimates and credible intervals). Mortality of infective larvae was generally 10 times lower than the mortality of the pre-infective stages. Estimates for infective mortality did not show a consistent pattern across temperature, with the lowest mortality at 25°C and highest mortality at 30°C. We were unable to estimate infective mortality at 35°C as there were no pre-infective parasites that developed to infective larvae at that temperature in any of the replicates. Development rates increased with temperature to a peak at 30°C, with a sharp decline to almost zero at 35°C (figure 5*b*).

The best-fit MTE model included an Arrhenius relationship for pre-infective mortality, constant infective mortality across temperatures of 0.022 (0.021, 0.023) d^−1^ and a Sharpe–Schoolfield relationship for development with an upper threshold at 30.6°C (electronic supplementary material, table A4). The best-fit MTE model, although simpler, had less predictive power than the model that estimated parameters in each temperature treatment independently (electronic supplementary material, table A2). Despite this, the MTE models have a major advantage in that they allow for prediction of mortality and development between and outside of the exact temperature treatments in experiments. Thus, in the host–parasite population modelling, we proceeded with using the MTE relationships.

There were two major limitations of using the estimated MTE parameters to predict mortality and development of free-living larvae on the tundra. First, *O. gruehneri* larvae will experience a much wider temperature range in nature than we considered in our experiments: ground temperatures along our simulated migration route were between −30.8°C and 25.7°C for the years 2000–2021. Thus, we had to make informed assumptions about the shape of the MTE curves at sub-zero temperatures, based on freezing survival of other Arctic-adapted parasites [67] (figure 5). Second, our ability to estimate upper thermal bounds on mortality of infective larvae from experimental data was limited because no larvae developed to the infective stage above 30°C in experiments. Although our experimental data suggested that the mortality of infective larvae was constant across temperatures, in the population modelling, we assume that this parameter follows a Sharpe–Schoolfield relationship with upper and lower thermal bounds that were difficult to estimate from experiments.

### 4.2. Host–parasite population modelling

#### 4.2.1. Annual dynamics under current conditions

**Figure 6.**
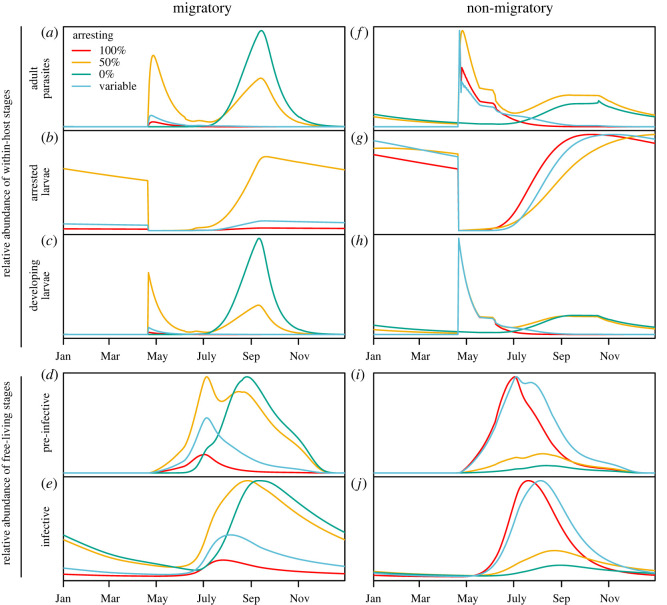
The relative abundance of parasites between simulations with migratory and non-migratory hosts (i.e. abundance scaled to the maximum parasites *within each arresting scenario*) over the last year of a 30-year simulation, assuming base case transmission of *β* = 10^−6^ d^−1^ (see electronic supplementary material, figures B5 and B6 for low and high transmission). Simulations including migration (left) or not (right) are shown, each under four scenarios for larval arrested development (colours; see legend).

Simulations showed strong annual cycles in the parasite burdens of hosts and in the density of free-living parasite larvae in the environment (figure 6). These annual cycles were driven largely by the annual pattern of egg output by adult parasites within hosts that predicted zero output of eggs through the winter months (electronic supplementary material, figure B2).

The timing of peak parasite burdens and effect of migration differed among the four scenarios for arrested development that we considered (figure 6). Under constant 100% arrested development, peak parasite burdens occurred in early May (figure 6*a,f*), driven by the resumption of larval development on 20 April with the onset of spring migration (figure 6*c,h*). The scenario with variable arresting, where an increasing proportion of larvae arrested as the summer progressed, showed a similar pattern through the year with the abundance of parasites in the environment peaking slightly later (figure 6*d,i*) due to contributions from larvae ingested early that spring and developed directly. This strategy led to a positive feedback between uptake of arresting larvae in the late summer and development and reproduction of parasites in the early spring that allowed parasite populations to eventually reach higher numbers (see §4.2.2 Long-term dynamics).

In both the 100% arresting and variable scenarios, parasite burdens were higher when host populations were non-migratory (i.e. setting the migration speed to 0 km d^−1^). Under 100% arresting, simulations of non-migratory host populations showed a 20-, 50- and 60-fold increase in the peak number of adult parasites, arrested larvae and developing larvae, respectively, compared with simulations with migratory hosts. Given that barren-ground caribou are migratory, and arrested development appears to be widespread [24], this suggests that the current system allows for migratory escape of host populations from *O. gruehneri* [72,73].

When parasites did not arrest but developed directly (0% arresting), parasite burdens tended to peak much later in early September and were higher in migratory hosts than in non-migratory hosts (figure 6*a,f*). Mixed strategies with 50% arresting led to an intermediate pattern with two peaks in parasite burdens and resulted in the second highest total infection pressure over the year (area under the curve in figure 6*e*) after the variable arresting scenario.

As expected, higher transmission rates led to higher parasite burdens within hosts, but did not alter the annual patterns described above (electronic supplementary material, figures B5 and B6). As we increased *β* by orders of magnitude, from 10^−7^ d^−1^ (low transmission) to 10^−6^ d^−1^ (base case) to 10^−5^ d^−1^ (high transmission), the adult parasite burdens within hosts also increased roughly by an order of magnitude. The abundance of infectious larvae in the environment changed less drastically, approximately doubling with each increase in *β*, emphasizing the higher efficiency of transmission at high *β*.

#### 4.2.2. Long-term dynamics

To understand the long-term host–parasite dynamics, we further summarized the annual patterns of infection (figure 6) by taking the annual average of daily mean adult parasite burdens per host each year, creating an annual metric of parasite pressure with units of parasites per host. Over longer simulations (200 years), we found that the annual average parasite burden and the total host population either reached a steady state or a two-point limit cycle with peaks in parasite pressure lagging behind peaks in host abundance as expected for density-dependent transmission processes. The transition from a single steady state to a periodic solution (i.e. a Hopf bifurcation) occurred as transmission rates increased (electronic supplementary material, figure B7).

**Figure 7.**
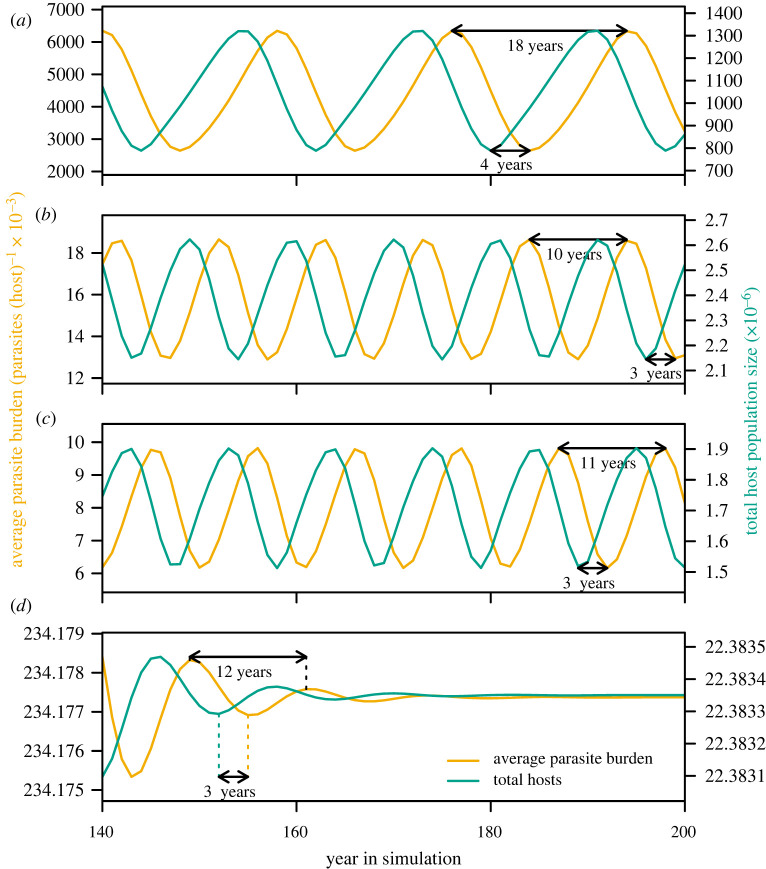
Cycles in the annual average parasite burden (calculated as the mean adult parasite burdens across all hosts averaged over the 365-day calendar year; yellow) and the total host population (sum of stationary and moving adults, yearlings and calves on 8 June each year; turquoise) for years 140–200 under four scenarios for larval arrested development: (*a*) 100% arresting, (*b*) 50% arresting, (*c*) 0% arresting and (*d*) variable arresting. See electronic supplementary material, figures B8 and B9 for cycles under low and high transmission.

The period of limit cycles depended on the propensity of larvae for arrested development. The period of cycles was longer under 100% arresting, with peaks occurring every 18 years (figure 7*a*). Under both 50% and 0% arresting, population cycles had a period of 10 years (figure 7*b,c*). Consistent with the different cycle periods, the peak parasite pressure lagged behind peak host abundance by approximately 4 years under 100% arresting and 2–3 years when there was 50% or 0% arresting.

When the propensity for arrested development increased throughout the summer, population cycles were dampened to long-term stable population sizes intermediate between 100% arresting and 50% or 0% arresting. However, cycles remained evident through the end of simulations under high transmission rates (electronic supplementary material, figure B9), suggesting that the transition from steady state to a two-point limit cycle (electronic supplementary material, figure B7) happens at different absolute transmission rates under different scenarios. Otherwise, patterns remained similar under the low and high transmission scenarios (electronic supplementary material, figures B8 and B9).

#### 4.2.3. Impact of temperature change

We summarized the effect of projected temperature changes on host–parasite dynamics by calculating the average (and s.d.) of annual average parasite burden and total host population over the last cycle period of the 200-year simulations for current temperatures and projected temperatures (both low and high emissions scenarios). The magnitude of these metrics differed substantially among the four scenarios for arrested development (table 2), with the highest parasite burdens and host population size under 100% arrested development followed by variable arrested development.

**Table 2.** The mean (s.d.) in annual average parasite burden and total host population size over the last cycle period of simulations (18, 10, 11 and 12 years for different arrested development scenarios; figure 7), comparing current climate and future temperatures under low emissions (RCP 2.6) and high emissions (RCP 8.5) scenarios.

arrested development	average parasite burdens (×10^−3^)			total host population (×10^−6^)		
	current	RCP 2.6	RCP 8.5	current	RCP 2.6	RCP 8.5
100%	4395.1 (1310.9)	4324.6 (1355.4)	4304.1 (1448)	1060.6 (186.1)	1011.3 (185.3)	951.7 (181.6)
50%	15.87 (2.14)	16.13 (2.12)	16.91 (2.14)	2.38 (0.17)	2.35 (0.17)	2.36 (0.16)
0%	7.97 (1.31)	8.12 (1.33)	8.24 (1.34)	1.71 (0.14)	1.67 (0.13)	1.75 (0.14)
variable	234.2 (0)	235.4 (0)	235.6 (0)	22.38 (0)	21.18 (0)	20.18 (0)

**Figure 8.**
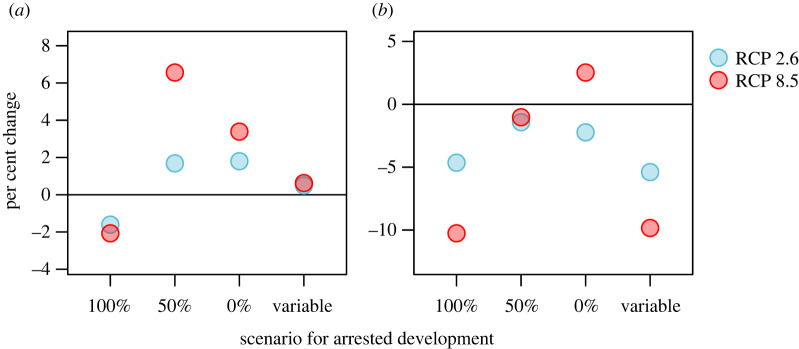
The per cent change in mean (*a*) annual average parasite burden and (*b*) total host population (table 2) under two climate change scenarios: low emissions (RCP 2.6) and high emissions (RCP 8.5). On the *x*-axis are the four scenarios for larval arrested development: (i) 100% arresting, (ii) 50% arresting, (iii) 0% arresting, and (iv) variable arresting (figure 3).

To compare the impact of climate change within a scenario, we calculated the per cent change in annual average parasite burden and total host population from those under the current climate. In general, parasite burdens were projected to increase and host populations to decrease, although there were differences among the different development scenarios (figure 8). Under 100% arresting and variable arresting, the per cent change in parasite burdens was minimal, but host populations were projected to decline by approximately 10% under RCP 8.5. Although it may seem counterintuitive that host populations decline when parasite burdens decrease (as with 100% arrested development), this can be explained by considering the timing of peak parasite burdens throughout the year. In the model, host populations are influenced by a decline in calf production with increasing parasite burdens in the previous autumn. Thus, even though the annual *average* parasite burden may not change (or even decline), faster development rates (electronic supplementary material, figure B11) led to a shift in the timing of peak parasite burdens to coincide with the period of impact on host populations in the autumn.

The only case that had increases in host populations was 0% arresting under the RCP 8.5 emissions scenario (figure 8*b*), although the increase was not substantial (table 2). In this case, the change in host population under RCP 2.6, however, was negative. Under both RCP 8.5 and RCP 2.6, annual average parasite burden increased due to a longer period for larval development (electronic supplementary material, figure B11B) that allowed for the parasite life cycle to be completed in a single summer with direct development. However, the mortality of free-living stages increased substantially more in the mid to late summer under RCP 8.5 than under RCP 2.6 (electronic supplementary material, figure B11D), due to the nonlinear change in mortality with increasing temperatures (table 2; figure 5). This meant that parasite burdens in the autumn, when host pregnancy rates were affected in our model, were lower under RCP 8.5, and thus host populations increased compared with the current temperature regime.

## 5. Discussion

Understanding the effect of climate change on host–parasite systems requires accounting for physiological and behavioural characteristics in multiple species, combining multiple, nonlinear pathways of effect. Mechanistic models are powerful tools for understanding the net outcomes and disentangling the contributing factors [10]. In this paper, we combined laboratory experiments and a spatial host–parasite population model to understand the potential impacts of climate change on migratory barren-ground caribou and their common nematode parasite, *O. gruehneri*.

We found that increasing temperatures due to climate change had very little impact on parasite burdens compared with aspects of both host and parasite behaviour. In contrast with general projections that disease risk is likely to increase with warming temperatures (e.g. [11]), we found that thermal responses of larval parasite development and mortality rates had a relatively small effect on host–parasite dynamics. Temperature-controlled laboratory experiments indicated increases in mortality and development rates of parasite larvae with warmer temperatures. When incorporated into the population model, these thermal responses led to an increase in annual average parasite burden with climate warming when parasites underwent direct development, as warming temperatures facilitated faster development, an extended transmission season and a shortened generation time. However, when we simulated 100% larval arrest in the caribou host, parasite burdens were minimally affected by temperature increases because arrested development prevented the parasite from shortening its generation time.

By contrast, parasite burdens differed by orders of magnitude depending on behaviour, increasing when all parasite larvae arrested their development and decreasing with migratory escape of hosts. These behavioural traits influence the spatio-temporal overlap of parasites and hosts—an aspect often ignored when making climate projections. The thermal performance of parasite larvae will affect the availability of parasite larvae to infect hosts, but the implications of that shift cannot be understood without considering other aspects of the host–parasite system that determine whether hosts are in the right place and time to become infected.

Long-term model simulations suggested that shifts in parasite life-history strategy from predominantly arrested development to direct development would have a larger impact on parasite burdens than changes in parasite thermal performance. Parasite burdens were orders of magnitude greater when parasite larvae arrested within hosts than under direct development, suggesting that a shift from arresting to direct development under warmer temperatures, as has been reported [24], would reduce parasite burdens. This can be understood because under direct development parasite larvae do not synchronously develop to reproductive adults in the early spring, but throughout the late summer and winter, a period when (i) egg shedding rates are low, (ii) any eggs that are shed are susceptible to extreme cold and associated mortality, and (iii) hosts migrate away before eggs can develop to infective-stage larvae. The associated reduction in parasite reproduction may potentially offset any increases due to higher thermal performance of parasite larvae. Further research is needed to determine if there is any fitness advantage of shifting to direct development under warmer temperatures, and what factors may affect the propensity for arresting in this parasite.

In addition to the life-history strategy of the parasite, host migration was a key component of the system, driving the locations and timing of transmission and ultimately determining the parasite burdens of hosts. Feedbacks between migration and parasitism are increasingly recognized to drive disease dynamics of migratory hosts [73], including in insects [74], fishes [75,76], birds [77,78] and ungulates [79,80]. As a result, parasitism has also been proposed as a potential driver of migratory behaviour [81] and migration may lead to the evolution of life-history traits in parasites, such as synchronized resumption of development after dormancy [24]. The importance of considering host movement for understanding and predicting wildlife disease risk cannot be overstated. More broadly, our results highlight how changes in behaviour, including migration and parasite dormancy, and not just thermal performance, are key to understanding the impacts of climate change on host–parasite systems.

In model simulations, host and parasite populations showed long-term cycles with periods from 10 to 20 years. Long-term cycles in barren-ground caribou populations have been suggested, with a period of 30–70 years that varies among herds [28,29]. The variables driving these fluctuations remain unknown, and there is probably a combination of extrinsic and intrinsic factors contributing, of which parasitism may be one [28,82]. We found that peaks in parasite burdens lagged behind peaks in host populations by 2–4 years, as theory would suggest when parasites negatively impact host survival and/or reproduction [83,84]. We observed longer lag times under 100% arrested development because two summers are required for parasites to complete their life cycle with arrested development and migration of hosts away from infective larvae.

The longer cycles and lag times under arrested development may also be explained because parasites had less of a regulatory effect on host populations in that scenario. Synchronous resumption of larval development within hosts in April meant that adult parasite burdens peaked relatively early in the year (late April–early May) and declined throughout the summer. Parasite burdens in the autumn were relatively low compared with other scenarios and had little impact on pregnancy rates. Thus, even though the peak adult parasite burdens were higher under 100% arresting, the timing of that peak meant that these high parasite burdens had minimal effect regulating the host population via impacts on reproduction, enabling the host (and parasite) populations to grow larger, over a longer period. This pattern points to arrested development as an example of host–parasite coevolution, maximizing the fitness of both, as well as an adaptation to a highly seasonal environment.

These results are probably sensitive to the assumptions we made about how parasites affect host population dynamics. In our model, pregnancy rates were negatively impacted by high parasite burdens during October prior to calving. This period of impact was chosen out of necessity: the only quantitative information on population-level effects of *Ostertagia* on *Rangifer* was from studies of semi-domesticated Svalbard reindeer, where parasite burdens were assessed in the autumn when animals were harvested [33,36]. In reality, *Ostertagia* infections probably have cumulative impacts through time on host body condition and, subsequently, reproduction. Although there are several studies on the relationship between *Ostertagia* burdens and body condition and pregnancy rates in free-ranging caribou populations (e.g. [34,35]), the results are not definitive, perhaps due to the relatively small sample sizes and the difficulty of assessing these impacts in free-ranging animals. Further, we only considered an impact of adult parasites, but Type II ostertagiasis associated with the synchronous development of arrested larvae in the spring is a well-documented disease of domesticated cattle, with acute impacts on hosts including severe inflammation, hypoproteinaemia and blood loss [85]. Integrating the impact on hosts of both larval and adult parasites over the entire season would more accurately reflect the biological mechanisms that lead to observed reductions in fecundity. Achieving this for the model we applied requires quantifying the relationships among cumulative parasite burdens, disease syndromes (i.e. Type II ostertagiasis), host body condition and fecundity—including any thresholds or nonlinear responses.

In addition to how parasites affect hosts, we had to make assumption about the demographic parameters of the parasites themselves. In particular, we applied parameters for the mortality rates of within-host parasite stages from a related species, *O. ostertagi*, which has relatively high mortality of adult parasites [63]. Cross-sectional studies of *O. gruehneri* infections in caribou and reindeer suggest that overwinter survival of adult parasites is high, with parasite burdens changing little between October and April [37,86]. Thus, the mortality rate of adult parasites may be lower than we assumed, which would increase overwinter survival of adult parasites and potentially dampen the annual cycles that we found.

Diverse physiological and behavioural responses to climate change illustrate the biological complexity of projecting climate change impacts on host–parasite systems. At a more basic level, however, are the uncertainties about the physical changes that will manifest in Arctic landscapes. We considered changes in temperature only. Even then, we were forced to make assumptions about how projected air temperature anomalies will affect ground temperatures, because projections for ground temperatures were not available. Climate models are becoming increasingly sophisticated, and future simulations may be able to predict how increasing air temperatures will affect ground temperatures, accounting for feedbacks due to thawing permafrost and changing snow cover. For example, decreased snow cover may reduce ground insulation over winter and result in colder temperatures experienced by larvae resulting in increased overwinter mortality of larvae in the environment. Earlier snowmelt and delayed onset of winter, however, will lengthen the window for larval development in the environment, potentially extending the transmission season.

Climatic processes may also influence host populations—a component that we did not address in this study. Climate change is predicted to alter the timing and duration of caribou migrations, as lakes and rivers thaw and extreme weather events become more frequent [87]. In some cases, migrations may halt altogether. For example, Dolphin and Union caribou migrate across sea ice from calving grounds on Victoria Island to their overwinter range on the mainland [57]. Later freeze-up in the autumn would lead to increased aggregation of hosts waiting to migrate south and could increase transmission during this period [73], which our model suggests could increase parasite burdens if there is some direct development. Plant communities are also responding to temperature change, which may increase the availability and quality of forage (reviewed in [32]) but also impact migration timing and parasite transmission dynamics.

Conserving wildlife species through the Anthropocene requires anticipating the multi-faceted impacts of climate change on ecosystems. Parasites are a key component of these ecosystems, with the potential to affect wildlife health and regulate host population abundance. We have shown that novel insights can arise from incorporating different pathways of impact using mechanistic population models. Specifically, we found that shifts in parasite life-history strategy and host migrations may supersede physiological effects of temperature on parasites predicted from laboratory experiments. These results emphasize the importance of considering multiple pathways of effect when predicting the impacts of climate change and may be used to focus future research and data collection.

## Data Availability

All data from laboratory experiments and code to estimate model parameters and reproduce population model simulations is available on GitHub at the following https://github.com/sjpeacock/OsterBou-pop, and have been archived within the Zenodo repository: https://doi.org/10.5281/zenodo.6637012. The data are provided in the electronic supplementary material [88].

## References

[1] Bush E, Lemmen DS. 2019 *Canada's changing climate report.* Ottawa, ON. See https://changingclimate.ca/CCCR2019/.

[2] Zhang X et al. 2019 Changes in temperature and precipitation across Canada. In Canada's changing climate report (eds E Bush, D Lemmen), pp. 112-193. Ottawa, ON: Government of Canada.

[3] Wassmann P, Duarte CM, Agustí S, Sejr MK. 2011 Footprints of climate change in the Arctic marine ecosystem. Glob. Chang. Biol. **17**, 1235-1249. (10.1111/j.1365-2486.2010.02311.x)

[4] Post E et al. 2009 Ecological dynamics across the Arctic associated with recent climate change. Science **325**, 1355-1358. (10.1126/science.1173113) 19745143

[5] Born D. 2019 Bearing witness? Polar bears as icons for climate change communication in *National Geographic*. Environ. Commun. **13**, 649-663. (10.1080/17524032.2018.1435557)

[6] Boonstra R et al. 2020 The stress of Arctic warming on polar bears. Glob. Chang. Biol. **26**, 4197-4214. (10.1111/gcb.15142) 32364624

[7] Kutz SJ, Hoberg EP, Molnár PK, Dobson A, Verocai GG. 2014 A walk on the tundra: host-parasite interactions in an extreme environment. Int. J. Parasitol. Parasit. Wildl. **3**, 198-208. (10.1016/j.ijppaw.2014.01.002) PMC4145143 25180164

[8] Dobson A, Molnár PK, Kutz S. 2015 Climate change and Arctic parasites. Trends Parasitol. **31**, 181-188. (10.1016/j.pt.2015.03.006) 25900882

[9] Brown JH, Gillooly JF, Allen AP, Savage VM, West GB. 2004 Toward a metabolic theory of ecology. Ecology **85**, 1771-1789. (10.1890/03-9000)

[10] Molnár PK, Sckrabulis JP, Altman KA, Raffel TR. 2017 Thermal performance curves and the metabolic theory of ecology—a practical guide to models and experiments for parasitologists. Parasitology **103**, 423-439. (10.1645/16-148) 28604284

[11] Harvell CD et al. 2002 Climate warming and disease risks for terrestrial and marine biota. Science **296**, 2158-2162. (10.1126/science.1063699) 12077394

[12] Kutz SJ, Hoberg EP, Polley L, Jenkins EJ. 2005 Global warming is changing the dynamics of Arctic host-parasite systems. Proc. R. Soc. B **272**, 2571-2576. (10.1098/rspb.2005.3285) PMC1559981 16321777

[13] Kutz SJ et al. 2013 Invasion, establishment, and range expansion of two parasitic nematodes in the Canadian Arctic. Glob. Chang. Biol. **19**, 3254-3262. (10.1111/gcb.12315) 23828740

[14] Kafle P, Peller P, Massolo A, Hoberg E, Leclerc LM, Tomaselli M, Kutz S. 2020 Range expansion of muskox lungworms track rapid arctic warming: implications for geographic colonization under climate forcing. Sci. Rep. **10**, 1-14. (10.1038/s41598-020-74358-5) 33057173 PMC7560617

[15] Kirk D, Jones N, Peacock S, Phillips J, Molnár PK, Krkošek M, Luijckx P. 2018 Empirical evidence that metabolic theory describes the temperature dependency of within-host parasite dynamics. PLoS Biol. **16**, e2004608. (10.1371/journal.pbio.2004608) 29415043 PMC5819823

[16] Molnár PK, Kutz SJ, Hoar BM, Dobson AP. 2013 Metabolic approaches to understanding climate change impacts on seasonal host-macroparasite dynamics. Ecol. Lett. **16**, 9-21. (10.1111/ele.12022) 23157563

[17] Kirk D, Luijckx P, Stanić A, Krkošek M. 2019 Predicting the thermal and allometric dependencies of disease transmission via the metabolic theory of ecology. Am. Nat. **193**, 661-676. (10.1086/702846) 31002572

[18] Hurford A, Cobbold CA, Molnár PK. 2019 Skewed temperature dependence affects range and abundance in a warming world. Proc. R. Soc. B **286**, 20191157. (10.1098/rspb.2019.1157) PMC6710591 31387510

[19] Paull SH, Johnson PTJ. 2014 Experimental warming drives a seasonal shift in the timing of host-parasite dynamics with consequences for disease risk. Ecol. Lett. **17**, 445-453. (10.1111/ele.12244) 24401007

[20] Molnár PK, Dobson AP, Kutz SJ. 2013 Gimme shelter – the relative sensitivity of parasitic nematodes with direct and indirect life cycles to climate change. Glob. Chang. Biol. **19**, 3291-3305. (10.1111/gcb.12303) 23801641

[21] Aleuy OA, Kutz S. 2020 Adaptations, life-history traits and ecological mechanisms of parasites to survive extremes and environmental unpredictability in the face of climate change. Int. J. Parasitol. Parasit. Wildl. **12**, 308-317. (10.1016/j.ijppaw.2020.07.006) PMC7569736 33101908

[22] Sommerville RI, Davey KG. 2002 Diapause in parasitic nematodes: a review. Can. J. Zool. **80**, 1817-1840. (10.1139/z02-163)

[23] Waller PJ, Rudby-Martin L, Ljungström BL, Rydzik A. 2004 The epidemiology of abomasal nematodes of sheep in Sweden, with particular reference to over-winter survival strategies. Vet. Parasitol. **122**, 207-220. (10.1016/j.vetpar.2004.04.007) 15219362

[24] Hoar BM, Eberhardt AG, Kutz SJ. 2012 Obligate larval inhibition of *Ostertagia gruehneri* in *Rangifer tarandus*? Causes and consequences in an Arctic system. Parasitology **139**, 1339-1345. (10.1017/S0031182012000601) 22953998

[25] Eysker M. 1993 The role of inhibited development in the epidemiology of *Ostertagia* infections. Vet. Parasitol. **46**, 259-269. (10.1016/0304-4017(93)90063-S) 8484216

[26] Kutz SJ, Jenkins EJ, Veitch AM, Ducrocq J, Polley L, Elkin B, Lair S. 2009 The Arctic as a model for anticipating, preventing, and mitigating climate change impacts on host-parasite interactions. Vet. Parasitol. **163**, 217-228. (10.1016/j.vetpar.2009.06.008) 19560274

[27] Joly K et al. 2019 Longest terrestrial migrations and movements around the world. Sci. Rep. **9**, 1-10. (10.1038/s41598-019-51884-5) 31654045 PMC6814704

[28] Gunn A. 2003 Voles, lemmings and caribou – population cycles revisited? Rangifer **23**, 105. (10.7557/2.23.5.1689)

[29] Bongelli E, Dowsley M, Velasco-Herrera VM, Taylor M. 2020 Do North American migratory barren-ground caribou subpopulations cycle? Arctic **73**, 326-346. (10.14430/arctic71029)

[30] Vors LS, Boyce MS. 2009 Global declines of caribou and reindeer. Glob. Chang. Biol. **15**, 2626-2633. (10.1111/j.1365-2486.2009.01974.x)

[31] COSEWIC. 2016 COSEWIC assessment and status report on the caribou *Rangifer tarandus*, barren-ground population, in Canada Committee on the Status of Endangered Wildlife in Canada. See http://www.registrelep-sararegistry.gc.ca/default.asp?lang=en&n=24F7211B-1.

[32] Mallory CD, Boyce MS. 2018 Observed and predicted effects of climate change on Arctic caribou and reindeer. Environ. Rev. **16**, 13-25. (10.1139/er-2017-0032)

[33] Stien A, Irvine RJ, Ropstad E, Halvorsen O, Langvatn R, Albon SD. 2002 The impact of gastrointestinal nematodes on wild reindeer: experimental and cross-sectional studies. J. Anim. Ecol. **71**, 937-945. (10.1046/j.1365-2656.2002.00659.x)

[34] Hughes J, Albon SD, Irvine RJ, Woodin S. 2009 Is there a cost of parasites to caribou? Parasitology **136**, 253-265. (10.1017/S0031182008005246) 19102793

[35] Steele J. 2013 The devil's in the diversity: divergent parasite faunas and their impacts on body condition in two Greenland caribou populations. Master's thesis, University of Calgary, Calgary, AB. (doi:10.11575/PRISM/28063). See http://hdl.handle.net/11023/420.

[36] Albon SD, Stien A, Irvine RJ, Langvatn R, Ropstad E, Halvorsen O. 2002 The role of parasites in the dynamics of a reindeer population. Proc. R. Soc. B **269**, 1625-1632. (10.1098/rspb.2002.2064) PMC1691070 12184833

[37] Carlsson AM, Justin Irvine R, Wilson K, Piertney SB, Halvorsen O, Coulson SJ, Stien A, Albon SD. 2012 Disease transmission in an extreme environment: nematode parasites infect reindeer during the Arctic winter. Int. J. Parasitol. **42**, 789-795. (10.1016/j.ijpara.2012.05.007) 22705063

[38] Hoar BM, Ruckstuhl K, Kutz S. 2012 Development and availability of the free-living stages of *Ostertagia gruehneri*, an abomasal parasite of barrenground caribou (*Rangifer tarandus groenlandicus*), on the Canadian tundra. Parasitology **139**, 1093-1100. (10.1017/S003118201200042X) 22717158

[39] Pandey VS. 1972 Effect of temperature on development of the free-living stages of *Ostertagia ostertagi*. J. Parasitol. **58**, 1037-1041. (10.2307/3278128) 4641869

[40] Rose H, Wang T, van Dijk J, Morgan ER. 2015 GLOWORM-FL: a simulation model of the effects of climate and climate change on the free-living stages of gastro-intestinal nematode parasites of ruminants. Ecol. Modell **297**, 232-245. (10.1016/j.ecolmodel.2014.11.033)

[41] Peacock SJ, Bouhours J, Lewis MA, Molnár PK. 2018 Macroparasite dynamics of migratory host populations. Theor. Popul. Biol. **120**, 29-41. (10.1016/j.tpb.2017.12.005) 29317230

[42] Peacock SJ, Krkošek M, Lewis MA, Molnár PK. 2020 A unifying framework for the transient parasite dynamics of migratory hosts. Proc. Natl Acad. Sci. USA **117**, 10 897-10 903. (10.1073/pnas.1908777117) PMC7245128 32358200

[43] Hubert J, Kerboeuf D. 1984 A new method for culture of larvae used in diagnosis of ruminant gastrointestinal strongylosis: comparison with fecal cultures. Can. J. Comp. Med. **48**, 63-71. 6713260 PMC1236007

[44] Russell DE, Whitfield PH, Cai J, Gunn A, White RG, Poole K. 2013 CARMA's MERRA-based caribou range climate database. Rangifer **33**, 145. (10.7557/2.33.2.2535)

[45] Leathwick DM. 2013 The influence of temperature on the development and survival of the pre-infective free-living stages of nematode parasites of sheep. N Z Vet. J. **61**, 32-40. (10.1080/00480169.2012.712092) 22913582

[46] Régnière J, Powell J, Bentz B, Nealis V. 2012 Effects of temperature on development, survival and reproduction of insects: experimental design, data analysis and modeling. J. Insect. Physiol. **58**, 634-647. (10.1016/j.jinsphys.2012.01.010) 22310012

[47] Régnière J, Powell J. 2003 Animal life cycle models. In Phenology: an integrative environmental science (ed. M Schwarz), pp. 295-315. Berlin, Germany: Springer Berlin.

[48] McCoy MW, Gillooly JF. 2008 Predicting natural mortality rates of plants and animals. Ecol. Lett. **11**, 710-716. (10.1111/j.1461-0248.2008.01190.x) 18422635

[49] Schoolfield RM, Sharpe PJH, Magnuson CE. 1981 Non-linear regression of biological temperature-dependent rate models based on absolute reaction-rate theory. J. Theor. Biol. **88**, 719-731. (10.1016/0022-5193(81)90246-0) 6790878

[50] Gunn A, Miller FL. 1986 Traditional behaviour and fidelity to caribou calving grounds by barren-ground caribou. Rangifer **1**, 151-158. (10.7557/2.6.2.640)

[51] Gunn A, Dragon J, Boulanger J. 2001 Seasonal movements of satellite-collared caribou from the Bathurst herd. Final report to the West Kitikmeot Slave Study Society. Yellowknife, Canada.

[52] McNeil P, Russell DE, Griffith B, Gunn A, Kofinas GP. 2005 Where the wild things are: seasonal variation in caribou distribution in relation to climate change. Rangifer **25**, 51. (10.7557/2.25.4.1770)

[53] The Bathurst Caribou Range Plan. 2018 Supporting report: caribou range assessment and technical information. Yellowknife, NT. See https://www.enr.gov.nt.ca/sites/enr/files/resources/draft_-_caribou_range_assessment_and_technical_information.pdf.

[54] Nagy JA. 2011 Use of space by caribou in northern Canada. University of Alberta. See https://era.library.ualberta.ca/items/98b7bd10-de1d-48f1-b681-66a0ca135ee4.

[55] Wilcove DS, Wikelski M. 2008 Going, going, gone: is animal migration disappearing? PLoS Biol. **6**, 1361-1364. (10.1371/journal.pbio.0060188) PMC2486312 18666834

[56] Mallory CD. 2019 Responses of Arctic caribou (*Rangifer tarandus*) to changing climate conditions. PhD thesis, University of Alberta, Canada.

[57] Poole KIMG, Gunn A, Patterson BR, Dumond M. 2010 Sea ice and migration of the Dolphin and Union caribou herd in the Canadian Arctic: an uncertain future. Arctic **63**, 414-428. (10.14430/arctic3331)

[58] Boulanger J, Gunn A, Adamczewski J, Croft B. 2011 A data-driven demographic model to explore the decline of the Bathurst caribou herd. J. Wildl. Manage. **75**, 883-896. (10.1002/jwmg.108)

[59] Thomas DC. 1982 The relationship between fertility and fat reserves of Peary caribou (*Rangifer tarandus pearyi*). Can. J. Zool. **60**, 597-602. (10.1139/z82-089)

[60] Pachkowski M, Côté SD, Festa-Bianchet M. 2013 Spring-loaded reproduction: effects of body condition and population size on fertility in migratory caribou (*Rangifer tarandus*). Can. J. Zool. **91**, 473-479. (10.1139/cjz-2012-0334)

[61] Armour J, Duncan M. 1987 Arrested larval development in cattle nematodes. Parasitol. Today **3**, 171-176. (10.1016/0169-4758(87)90173-6) 15462948

[62] Hoar BM. 2012 Ecology and transmission dynamics of *Ostertagia gruehneri* in barrenground caribou. See https://prism.ucalgary.ca/handle/11023/289.

[63] Grenfell BT, Smith G, Anderson RM. 1987 A mathematical model of the population biology of *Ostertagia ostertagi* in calves and yearlings. Parasitology **95**, 389-406. (10.1017/S0031182000057826) 3696772

[64] Shaw DJ, Grenfell BT, Dobson AP. 1998 Patterns of macroparasite aggregation in wildlife host populations. Parasitology **117**, 597-610. (10.1017/S0031182098003448) 9881385

[65] Anderson RM, May RM. 1978 Regulation and stability of host-parasite population interactions: I. Regulatory processes. J. Anim. Ecol. **47**, 219-247. (10.2307/3933)

[66] Stien A, Irvine RJ, Langvatn R, Albon SD, Halvorsen O. 2002 The population dynamics of *Ostertagia gruehneri* in reindeer: a model for the seasonal and intensity dependent variation in nematode fecundity. Int. J. Parasitol. **32**, 991-996. (10.1016/S0020-7519(02)00071-1) 12076628

[67] Aleuy OA, Peacock S, Hoberg EP, Ruckstuhl KE, Brooks T, Aranas M, Kutz S. 2020 Phenotypic plasticity and local adaptation in freeze tolerance and its implications for parasite dynamics in a changing world: the case of *Marshallagia marshalli*. Int. J. Parasitol. **50**, 161-169. (10.1016/j.ijpara.2019.12.004) 32004511

[68] Convey P, Coulson SJ, Worland MR, Sjöblom A. 2018 The importance of understanding annual and shorter-term temperature patterns and variation in the surface levels of polar soils for terrestrial biota. Polar Biol. **41**, 1587-1605. (10.1007/s00300-018-2299-0)

[69] Cleveland WS, Grosse E, Shyu WM. 1992 Chapter 8: Local regression models. In Statistical models *in S* (eds JM Chambers, TJ Hastie). New York, NY: Routledge. (doi:10.1201/9780203738535)

[70] R Core Team. 2021 R: a language and environment for statistical computing. Vienna, Austria: R Foundation for Statistical Computing. See https://www.R-project.org/.

[71] Amman C, Boehnert J, Wilhelmi O. 2018 *World Climate Data CMIP5* *Multi Model Ensemble*. Boulder, CO: Research Applications Laboratory, National Center for Atmospheric Research. See https://learn.arcgis.com/en/projects/explore-future-climate-projections/ (accessed 12 July 2021).

[72] Folstad I, Nilssen AC, Halvorsen O, Andersen J. 1991 Parasite avoidance: the cause of post-calving migrations in *Rangifer*? Can. J. Zool. **69**, 2423-2429. (10.1139/z91-340)

[73] Altizer S, Bartel R, Han BA. 2011 Animal migration and infectious disease risk. Science **331**, 296-302. [cited 2014 May 29] (10.1126/science.1194694) 21252339

[74] Bartel RA, Oberhauser KS, De Roode JC, Altizer SM. 2011 Monarch butterfly migration and parasite transmission in eastern North America. Ecology **92**, 342-351. (10.1890/10-0489.1) 21618914 PMC7163749

[75] Krkošek M, Gottesfeld A, Proctor B, Rolston D, Carr-Harris C, Lewis MA. 2007 Effects of host migration, diversity and aquaculture on sea lice threats to Pacific salmon populations. Proc. R. Soc. B **274**, 3141-3149. (10.1098/rspb.2007.1122) PMC2293942 17939989

[76] Poulin R, Closs GP, Lill AWT, Hicks AS, Herrmann KK, Kelly DW. 2012 Migration as an escape from parasitism in New Zealand galaxiid fishes. Oecologia **169**, 955-963. (10.1007/s00442-012-2251-x) 22271201

[77] Van Dijk JGB, Hoye BJ, Verhagen JH, Nolet BA, Fouchier RAM, Klaassen M. 2014 Juveniles and migrants as drivers for seasonal epizootics of avian influenza virus. J. Anim. Ecol. **83**, 266-275. (10.1111/1365-2656.12131) 24033258 PMC3869896

[78] Emmenegger T, Bauer S, Dimitrov D, Olano Marin J, Zehtindjiev P, Hahn S. 2018 Host migration strategy and blood parasite infections of three sparrow species sympatrically breeding in Southeast Europe. Parasitol. Res. **117**, 3733 –3741. (10.1007/s00436-018-6072-7) 30232606

[79] Teitelbaum CS, Huang S, Hall RJ, Altizer S. 2018 Migratory behaviour predicts greater parasite diversity in ungulates. Proc. R. Soc. B **285**, 20180089. (10.1098/rspb.2018.0089) PMC5897646 29563269

[80] Morgan ER, Medley GF, Torgerson PR, Shaikenov BS, Milner-Gulland EJ. 2007 Parasite transmission in a migratory multiple host system. Ecol. Model **200**, 511-520. (10.1016/j.ecolmodel.2006.09.002)

[81] Shaw AK, Binning SA. 2016 Migratory recovery from infection as a selective pressure for the evolution of migration. Am. Nat. **187**, 491-501. (10.1086/685386) 27028077

[82] Hudson PJ, Dobson AP, Newborn D. 1998 Prevention of population cycles by parasite removal. Science **282**, 2256-2258. (10.1126/science.282.5397.2256) 9856948

[83] Anderson RM. 1980 Depression of host population abundance by direct life cycle macroparasites. J. Theor. Biol. **82**, 283-311. (10.1016/0022-5193(80)90104-6) 7374181

[84] May RM, Anderson RM. 1978 Regulation and stability of host-parasite population interactions: II. Destabilizing processes. J. Anim. Ecol. **47**, 249-267. (10.2307/3934)

[85] Anderson N. 1988 Aspects of the biology of *Ostertagia ostertagi* in relation to the genesis of ostertagiasis. Vet. Parasitol. **27**, 13-21. (10.1016/0304-4017(88)90057-X) 3284162

[86] Irvine RJ, Stien A, Halvorsen O, Langvatn R, Albon SD. 2000 Life-history strategies and population dynamics of abomasal nematodes in Svalbard reindeer (*Rangifer tarandus platyrhynchus*). Parasitology **120**, 297-311. (10.1017/S0031182099005430) 10759088

[87] Mallory CD, Williamson SN, Campbell MW, Boyce MS. 2020 Response of barren-ground caribou to advancing spring phenology. Oecologia **192**, 837-852. (10.1007/s00442-020-04604-0) 31982951

[88] Peacock SJ, Kutz SJ, Hoar BM, Molnár PK. 2022 Behaviour is more important than thermal performance for an Arctic host–parasite system under climate change. Figshare. (10.6084/m9.figshare.c.6135956) PMC9399711 36016913

